# Claws in the Capital: Human–Leopard Conflict Hotspots and Community Perceptions in Kathmandu Valley, Nepal

**DOI:** 10.1002/ece3.72678

**Published:** 2025-12-16

**Authors:** Pratistha Shrestha, Khem Lal Bishwakarma, Rupesh Maharjan, Dipesh Kumar Sharma

**Affiliations:** ^1^ Central Department of Zoology, Institute of Science and Technology Tribhuvan University Kirtipur Bagmati Nepal; ^2^ Government of Nepal, Ministry of Forests and Environment Forest Research and Training Centre (FRTC) Kathmandu Bagmati Nepal; ^3^ Technical University Dresden (TUD) Institute of International Forestry and Forest Products Tharandt Germany; ^4^ Caesar Kleberg Wildlife Research Institute Texas A & M University Kingsville Texas USA; ^5^ Département de Biologie, Chimie et géographie Université du Québec à Rimouski Rimouski QC Canada

**Keywords:** anthropogenic factors, coexistence, conservation, MaxEnt, Shivapuri Nagarjun National Park (SNNP)

## Abstract

In areas where forests and human‐dominated landscapes intersect, humans and wildlife compete or collide in their efforts to utilize natural resources. The Kathmandu Valley hosts a suitable habitat for several wildlife species, including the leopard ( *Panthera pardus* ), which inhabits the surrounding forest patches. Over the past few years, there has been an increase in human–leopard interactions that have raised concern for the increase in human–leopard conflicts. To better understand and visualize the conflict within the valley, this study modeled human–leopard conflict using leopard conflict data and environmental variables influencing those conflicts. A human–leopard conflict hotspot map was generated using the MaxEnt modeling approach, identifying the high‐risk zones primarily near forest edges and expanding settlements. The analysis highlights key factors, such as canopy cover, the human influence index, slope, and proximity to water bodies, influencing human–leopard conflict. Additionally, ordinal logistic regression was used to understand people's attitudes towards leopards, which remained largely positive despite rising conflicts between humans and leopards. The results showed an encouraging sign for governmental bodies seeking to mitigate conflicts through targeted, individual‐level awareness programs in the near future.

## Graphical abstract

Over the past few years, the increase in human–leopard interactions in the Kathmandu Valley has raised concerns about the rise in human–leopard conflicts. This study highlights key factors, such as canopy cover, the human influence index, slope, and proximity to water bodies that influence human–leopard conflict. Despite rising conflicts, public attitudes remain largely positive, suggesting that targeted awareness programs can be effective in mitigating future conflicts.

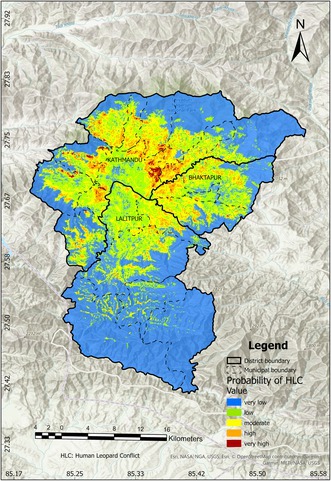

## 1 Introduction

Human–carnivore conflicts arise when the behaviors and requirements of carnivores adversely affect the objectives of humans and vice versa. These conflicts often stem from competition for shared resources, particularly space and food. The changes in the landscape and land use for structural development like roads (Maharjan, Langbein, et al. 2025; Pandey et al. 2025) and livelihood of the local community (Semwal et al. 2004; Pandey et al. 2024) undoubtedly have effects on the home range and movement of carnivores (Treves and Karanth 2003; Chapron and López‐Bao 2014; Dickman et al. 2014; Karki and Rawat 2014; DNPWC 2017). In human‐dominated areas, carnivores are often regarded as undesirable, which leads to negative interactions between them (Bagchi and Mishra 2006; Dar et al. 2009; Inskip and Zimmermann 2009; Athreya et al. 2011). The leopard ( *Panthera pardus* ), a widely distributed carnivore in Nepal from lowland Terai to high mountains up to 4400 m asl (DNPWC 2017), requires a wide range and contiguous suitable habitat (Dickman and Marker 2005; DNPWC 2017) at a distance from human settlements. Thus, the conflict between humans and leopards arises across the habitat range of the latter (Balme et al. 2007; Stein et al. 2016) in the form of livestock depredation, retaliatory killing, human injury and casualty, and property damage (DNPWC 2017).

The spatiotemporal patterns of all human–wildlife conflict are poorly documented across the Indian subcontinent, including Nepal (Lamichhane et al. 2023). Thus, using the physical conflict locations for spatial risk modeling could be a practical method for future planning on reducing human–leopard conflicts (HLCs) by predicting and mapping conflict hotspots (Lamichhane et al. 2023; Sharma et al. 2020; Treves et al. 2011; Miller 2015; Ruda et al. 2018). While location points can be accessed through secondary sources such as media reports, governmental documents, and confirmation by concerned authorities, extracting consistent covariate data of each associated conflict point occurring sparsely over different time periods is challenging. Therefore, in the context of HLC, the global remote sensing data are critical for predicting conflict hotspots where human expansion overlaps with wildlife habitat, leading to increased encounters and conflicts (Miller 2015; Kitratporn and Takeuchi 2019; Sharma et al. 2020; Egri et al. 2022).

Covariates extracted from remote sensing devices include geographical data, climatic data, distance to different land cover types, and the human influence index (HII) of the study area. While climate influences the natural species distribution range, topographic factors like elevation and terrain also influence the dynamics of conflict. Rugged landscapes, being less accessible to humans, create a natural barrier, ultimately influencing the nature and intensity of human–wildlife interactions in unexpected ways (Neilson et al. 2013; Aryal et al. 2014). In the areas vulnerable to climate change, wildlife's habitat composition, availability, and accessibility of resources would be greatly impacted, ultimately affecting the conflict (Mallick 2012; Sapkota and Rijal 2016; Cushman et al. 2018). Land use and land cover data can emphasize the main anthropogenic disturbances such as deforestation, human encroachment, land conversion, and fragmentation, affecting wildlife movement within their habitat and bringing them closer to human settlements (Raubenheimer et al. 2016; Acharya et al. 2017; Maharjan, Young, et al. 2025; Pandey et al. 2025). Similarly, the HII, an index incorporating human population density, land use pattern, infrastructure, night light, and human access, can quantitatively measure anthropogenic pressure on the environment (Sanderson et al. 2002). For prioritizing conflict mitigation strategies and conservation efforts, it is essential to identify areas with elevated risk of conflict, understanding its cause, and analyzing potential drivers. This helps to strategically plan for mitigating the conflict by utilizing several measures such as the establishment of buffer zones and wildlife corridors, and the implementation of community education programs to inform management strategies (Treves et al. 2011; Miller 2015; Miller et al. 2015; Broekhuis et al. 2017; Ghoddousi et al. 2020).

Prior to this study, insights from spatially explicit maps of HLC have been provided from the eastern Himalayas (Naha et al. 2020; Sharma et al. 2020), western Himalayas (Naha et al. 2020), and western mid‐hill regions (Lamichhane et al. 2023) of Nepal. However, conflict maps for mitigation strategies have not yet been developed for the Kathmandu Valley, where encounters with leopards in residential areas and predation on domestic livestock have been escalating due to rapid human population growth and increasing encroachment on leopard habitats (Koirala et al. 2012; Bhandari and Thapa 2015). In such cases, predictive models for conflict distribution mapping are an inevitable tool, which can be used in planning and prioritizing mitigation strategies. In the current scenario of ever‐growing machine learning algorithms, the Maximum Entropy (MaxEnt) model (Phillips et al. 2017) is more suitable and accurate for conflict mapping, which only uses presence locations of the target species (Sharma et al. 2020; Yadav et al. 2021; Lamichhane et al. 2023).

The most obvious source of HLC is the direct impact on livestock, exerting fear and significant economic loss. The real impact of wildlife damage, and the level of antagonism created, depends to some extent upon the relative wealth and security of the people affected (Zimmermann et al. 2001; Røskaft et al. 2007; Kansky and Knight 2014). People rarely respond to wildlife damage in a purely rational way; instead, they are strongly influenced by species biology, cultural norms, antagonism, political attitudes, and management practices (Athreya and Belsare 2007). On an individual level, people's responses are best explained by gender, education level, age, fear, and belief (Dickman 2012; Suryawanshi et al. 2014). Negative perceptions, driven by fear of livestock loss or safety concerns, can lead to retaliatory actions, such as killing or harming wildlife; whereas positive or neutral attitudes can promote tolerance and foster collaboration in conservation initiatives (Suryawanshi et al. 2014). Although several studies (Maharjan 2017; Kunwar and Koju 2019; Dhungana et al. 2022; Tiwari et al. 2024) have explored people's attitudes towards leopard conservation and habitat management, they mainly used descriptive analysis and hypothesis testing without employing regression‐based analysis, which makes it difficult to fully understand people's perceptions towards leopard conservation. Thus, this study has employed ordinal logistic regression to analyze people's attitudes towards leopard conservation.

The hotspot mapping of conflict‐prone areas within the Kathmandu Valley, along with addressing people's perceptions, provides a more complete knowledge of the fundamental causes and trends of conflict that helps to customize mitigating measures not just to the location and timing of conflicts but also to how local populations view and handle them. These strategies improve the success of conservation initiatives by matching community involvement with conflict management, therefore producing more locally supported solutions, ensuring that conservation efforts are proactive rather than reactive (Miller et al. 2016). Therefore, to provide valuable information for future conflict resolution interventions and assure the long‐term survival of the leopard population, this study aims to produce a reliable HLC hotspot map based on verified conflict incident records and assess people's attitudes towards the leopard in the Kathmandu Valley. The objective of the study was to (i) find important environmental variables influencing the HLC and (ii) identify the major target group of intervention that can play a crucial role in minimizing HLCs in the Kathmandu Valley.

## 2 Material and Methods

### 2.1 Study Area

**FIGURE 1:**
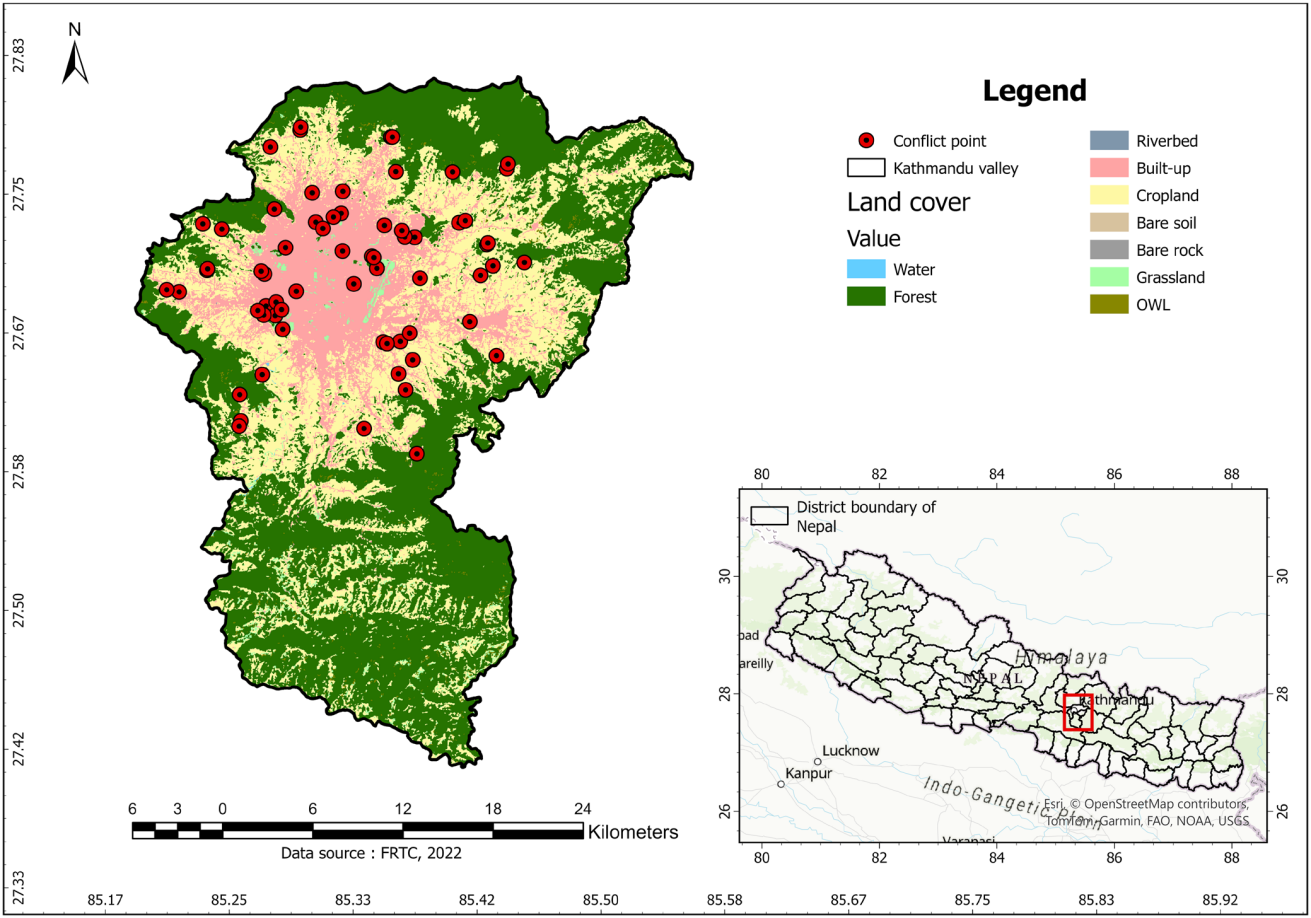
A map of the Kathmandu Valley located in Central Nepal, consisting of three districts—Kathmandu, Lalitpur, and Bhaktapur—depicting land cover types and human–leopard conflict points within the valley, collected from secondary sources.

Kathmandu Valley, located in central Nepal, encompasses three districts: Kathmandu, Bhaktapur, and Lalitpur and is situated at an elevation of 1350 m asl (27°32′13″–27°49′10″N and 85°11′31″–85°31′38″E) (Thapa et al. 2008; Pant and Dongol 2009) (Figure 1). The region has a humid subtropical climate, with temperatures ranging from a maximum of 23.9°C in June to a minimum of 4.9°C in January, and an average annual rainfall of 2812 mm (Climate‐Data 2022). Surrounding the valley, there are four mountain ranges: Shivapuri (2800 m), Nagarjun (2825 m), Phulchowki (2795 m), and Chandragiri (2551 m). Within the highly urbanized valley, the Shivapuri Nagarjun National Park (SNNP) is home to a rich diversity of wildlife, including protected species such as the Clouded leopard ( *Neofelis nebulosa* ), Chinese pangolin ( *Manis pentadactyla* ), Assamese monkey ( *Macaca assamensis* ), Leopard cat ( *Prionailurus bengalensis* ), and Leopard ( *Panthera pardus* ) (Poudyal et al. 2023; SNNP 2024).

### 2.2 Data Collection

#### 2.2.1 Spatial Pattern of HLC

Conflict data was collected from secondary sources, including media reports (national and local newspapers) and internet searches using various keyword combinations such as “Kathmandu,” “leopard attack,” “conflict,” “injured” to document the presence of leopards in the human‐dominated landscape of Kathmandu Valley, from 2006 to 2024. The collected secondary data were verified through field visits and interviews with local residents about the reliability of the reported incidents. After screening and removing spatially autocorrelated data (Veloz 2009), 55 out of the 83 GPS coordinates were retained as conflict points for modeling and mapping HLC hotspots. Although the sample size is relatively limited, it represents the available verified cases within the study period and area, which is typical for studies addressing rare and spatially constrained conflict events.

### 2.3 Predictor Variables

Certain environmental factors, such as temperature, precipitation, and canopy cover, are typically associated with species distribution; however, they can also influence the spatial pattern of human–wildlife conflict (Fidino et al. 2022; Abrahms et al. 2023). These variables affect vegetation growth and prey availability, which in turn shape leopard movement, hunting behavior, and habitat selection. For example, areas with high canopy cover may provide both prey and concealment opportunities for leopards, increasing the likelihood of encounters near human settlements (Gupta et al. 2017; Sharma et al. 2020). Similarly, variation in temperature and precipitation can alter habitat suitability and prey distribution, indirectly influencing conflict risk. Additionally, geographic factors, such as the proximity to resources or human settlement areas, can affect the likelihood of interactions between leopards and humans (Athreya et al. 2013; Mondal et al. 2013; Ramesh et al. 2020; Rather et al. 2020; Majumder et al. 2024). The HII, which considers population density, land use, infrastructure, and light pollution, can serve as a valuable indicator of how much human activity has altered the landscape, potentially leading to competition for space and resources. By shaping where leopards and humans overlap, these factors act as important predictors of conflict occurrence.

### 2.4 MaxEnt Model

Maxent modeling is a widely used machine learning tool for species distribution modeling (SDM), particularly effective when working with presence‐only data. Although it needs presence‐only data, it takes into account the pseudo‐absences and is different from other algorithms such as Bioclim or Surface Range Envelope (SRE), the Mahalanobis Distance, the Domain algorithm, and the Environmental Niche Factor Analysis (ENFA), which account for presence‐only data and don't consider absences (Gobeyn et al. 2019). It estimates the probability of a species' presence across a landscape by finding the distribution of maximum entropy, subject to constraints defined by environmental variables (Phillips et al. 2006).

For this study, environmental predictor variables, including topographic, bioclimatic, and anthropogenic factors, derived from global remote sensing datasets (Table 1), were chosen based on their relevance to leopard habitat preferences and HLC occurrences. Topographic variables (elevation, slope, aspect) describe terrain complexity influencing the ranging pattern of large carnivores (Powell and Mitchell 1998; Husseman et al. 2003). Bioclimatic variables (temperature, precipitation, NDVI, canopy cover) reflect habitat productivity and vegetation structure, which affect prey availability and cover (Pettorelli et al. 2005; Carthew et al. 2013; Rendall et al. 2021; Pandey et al. 2025). Anthropogenic predictors (HII, cropland proximity, and distance to water bodies) represent human presence and land‐use intensity that may increase spatial overlap between humans and carnivores (Grilo et al. 2009; Özcan and Özkazanç 2020).

**TABLE 1:** Selected variables for hotspot distribution modeling using Maxent after checking for multicollinearity.

Variables (unit)	Category	Original resolution	Sources/software
Slope (°)	Topographic	30 m × 30 m	SRTM (earthdata.nasa.gov)
Aspect (°)	Topographic	30 m × 30 m	SRTM (earthdata.nasa.gov)
NDVI	Environmental	250 m × 250 m	MODIS/061/MOD13Q1
Canopy cover (%)	Environmental	30 m × 30 m	https://lcluc.umd.edu/
BIO 4 (temperature seasonality, °C)	Environmental	1 km × 1 km	worldclim.org
BIO 6 (Min temperature of coldest month, °C)	Environmental	1 km × 1 km	worldclim.org
BIO 9 (mean temperature of driest quarter, °C)	Environmental	1 km × 1 km	worldclim.org
BIO 13 (precipitation of wettest month, mm)	Environmental	1 km × 1 km	worldclim.org
BIO 14 (precipitation of driest month, mm)	Environmental	1 km × 1 km	worldclim.org
BIO 16 (precipitation of wettest quarter, mm)	Environmental	1 km × 1 km	worldclim.org
Distance from water bodies (m)	Geographic	30 m × 30 m	https://www.hydrosheds.org/ + ArcGIS pro
Distance from cropland (m)	Geographic	30 m × 30 m	FRTC 2022 + ArcGIS pro
Distance from grassland (m)	Geographic	30 m × 30 m	FRTC 2022 + ArcGIS pro
Distance from OWL (m)	Geographic	30 m × 30 m	FRTC 2022 + ArcGIS pro
Human Influence Index (HII)	Anthropogenic		https://sedac.ciesin.columbia.edu/

The 30 variables were further checked for multicollinearity for selecting variables based on the variance inflation factor (vif) < 10, using packages such as *usdm* (Marquardt 1970; Kaky et al. 2020; Naimi 2023) and *dismo* (Hijmans et al. 2017), and only 15 important variables were chosen and resampled to 30 m × 30 m resolution to run the model using the *maxnet* package v.3.4.3 (Phillips 2022) in R‐studio v.4.3.3 (R Core Team 2024).

The occurrence data were split into 80% training and 20% test datasets. Additionally, 10‐fold cross‐validation with 25 replications was used to evaluate the model's robustness and reliability. To simulate absence points, 10,000 background points were generated. The model's performance was assessed using the Area Under the Receiver Operating Characteristic Curve (AUC), with a higher AUC value, approaching 1, indicating better predictive performance (Jiménez‐Valverde 2012). The jackknife test was done to evaluate the relative contribution of single environmental variables in the model (Shcheglovitova and Anderson 2013; Yuan et al. 2015).

The hotspot map was generated at a 30 m × 30 m resolution ranging from 0 to 1, values near to 1 meaning higher probability of conflicts. The map was then reclassified into five categories in ArcMap version 10.8 (ESRI 2021) viz. very low (0–0.2), low (0.2–0.4), moderate (0.4–0.6), high (0.6–0.8), and very high (0.8–1) risk zones, based on the classification reference of previous studies (Yang et al. 2013; Qin et al. 2017; Jha and Jha 2021). Afterwards, the areas for high and very high‐risk zones, i.e., probability between 0.6 and 1, were calculated.

### 2.5 People's Perception

#### 2.5.1 Sampling Design

A two‐phase purposive cluster sampling design was used for data collection. After verifying and removing overlapping records from media reports, zoo records (2012–2016), and other government sources, 29 clusters were identified as the target population. Out of these, 21 clusters were randomly selected for structured interviews with local residents. In total, 185 households (HHs) were surveyed, using a one‐in‐five household sampling approach, with one individual from each household interviewed in areas around human–leopard conflict hotspots. The number of households selected for interviews was allocated based on the proportionate weight of conflict incidents in the respective districts.

To quantify local people's attitudes, structured interviews were conducted based on five questions that were scored and summed together (Table S1). Short, simple questions were drafted to minimize the risk of interviewee fatigue and disinterest, and the questionnaire length was kept concise (Morina 2015). The questions were designed with a limited set of possible responses to maintain clarity. Interviews were conducted in the Nepali language to facilitate communication. The formal interview began by determining whether the interviewee could differentiate between the leopard and other similar‐looking leopards using photographs. In total, 185 individuals across 21 sample clusters in Kathmandu Valley were interviewed, all of whom correctly identified the leopards in the photographs. Four control variables (gender, age, location, and education level) were quantified and analyzed, as these factors could influence people's attitudes.

#### 2.5.2 Ordinal Logistic Regression

Ordinal logistic regression, a statistical method suitable for analyzing categorical response data with a natural order, was employed to analyze respondents' attitudes towards the leopard. Scores ranged from −2 (most negative) to +2 (most positive), with attitudes categorized as negative (< −1), neutral (−1 to 1), and positive (> 1), following the classification by Suryawanshi et al. (2014). A 5‐point Likert scale was not used considering respondents' difficulty to distinguish between “agree” and “strongly agree,” as well as “disagree” and “strongly disagree”. The model examined how various factors such as Gender (male and female), Age (18–35 years, 35–55 years, 55–70 years, and greater than 70 years old), Education (higher: university degree, intermediate: up to high school, and lower: illiterate to up to 5 grade) influenced people's perspectives. The model and its performance were evaluated using likelihood ratio tests based on chi square statistics. The odds ratio (OR) was used to represent the magnitude and direction of the effect, indicating the change in the odds of the outcome for a unit change in the predictor variable (Auster et al. 2020; Dhungana et al. 2022). For each estimated odds ratio, the 95% confidence intervals (CIs) were calculated to assess the precision and statistical significance. The analysis was implemented using the *MASS* package (Ripley et al. 2024) in R‐Studio v. 4.3.3 (R Core Team 2024).

## 3 Results

### 3.1 Hotspot Map of HLC in Kathmandu Valley

**FIGURE 2:**
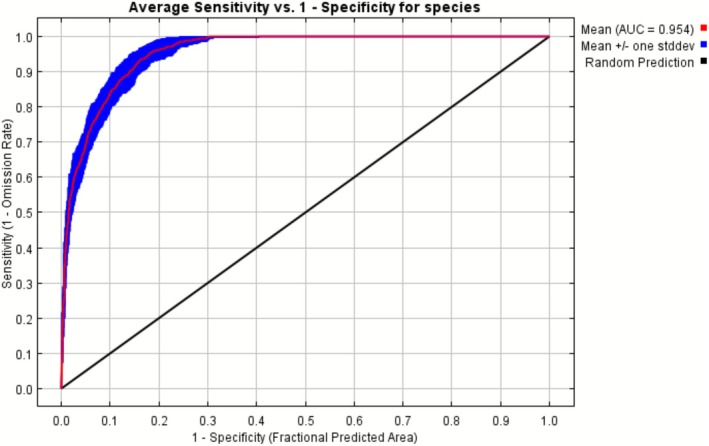
The estimated area under the curve (AUC) for the training data in the MaxEnt model with 25 replicates for human–leopard conflict, where the red line denotes the mean AUC value, and the blue area represents ±1SD.

**FIGURE 3:**
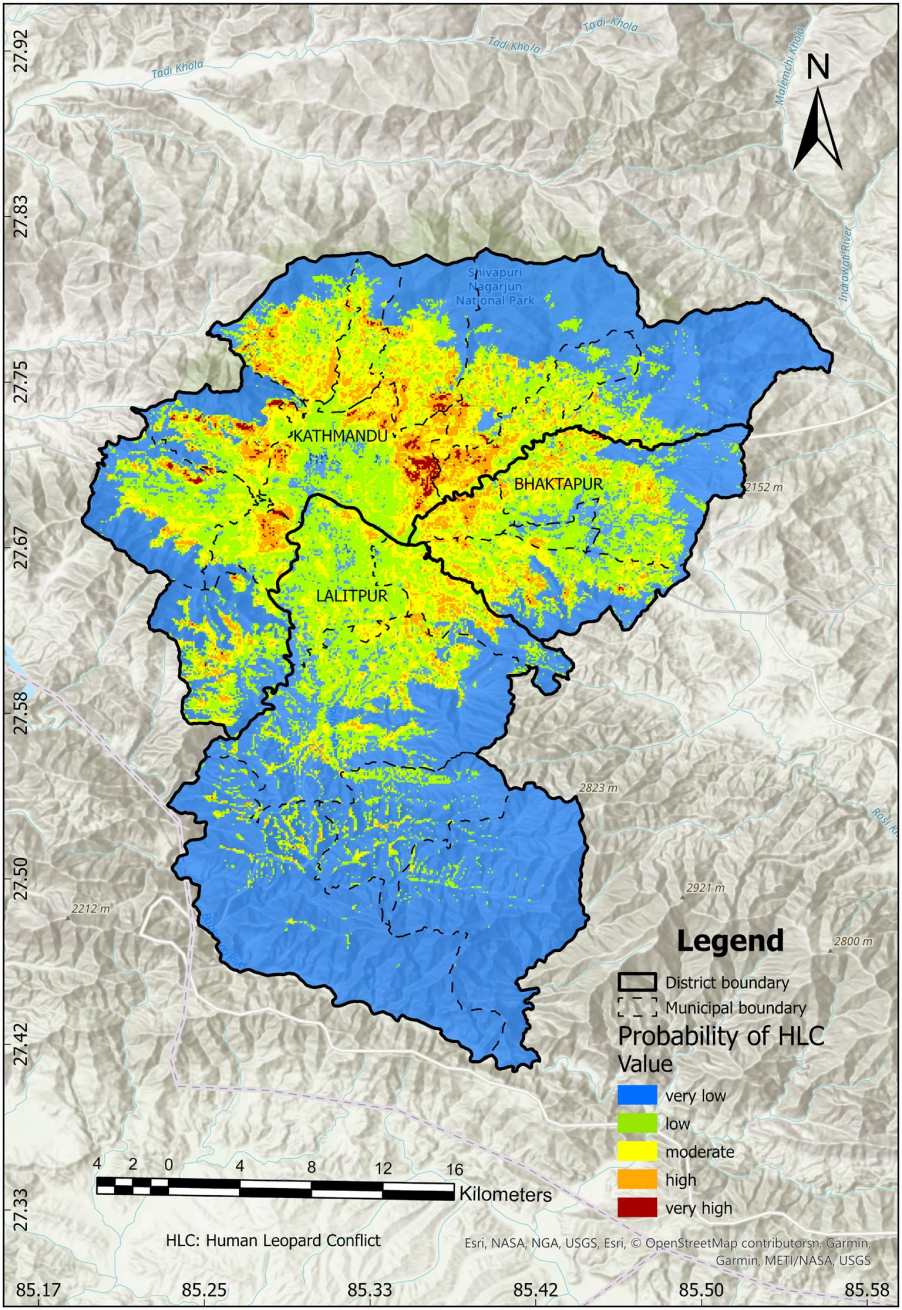
Human–leopard conflict hotspot map of Kathmandu Valley showing district and municipality boundaries.

A total of 55 HLC locations were identified in the Kathmandu valley, based on verified conflict incidents from 2006 to 2024, primarily recorded from media reports, Central Zoo records, government records, and journal articles. The model indicated strong predictive performance for HLC areas, with an AUC of 0.954 on the training set and 0.881 on the test set (Figure 2). The hotspot map, generated at a 30 m × 30 m resolution and ranging from 0 to 1, shows that conflicts are distributed across the entire valley (Figure 3). Areas with the highest probability of conflict (> 0.6) cover a total of 55.33 km^2^, including 5.76 km^2^ classified as very high‐risk areas (probability > 0.8).

### 3.2 Factors Influencing Conflict Occurrence

**FIGURE 4:**
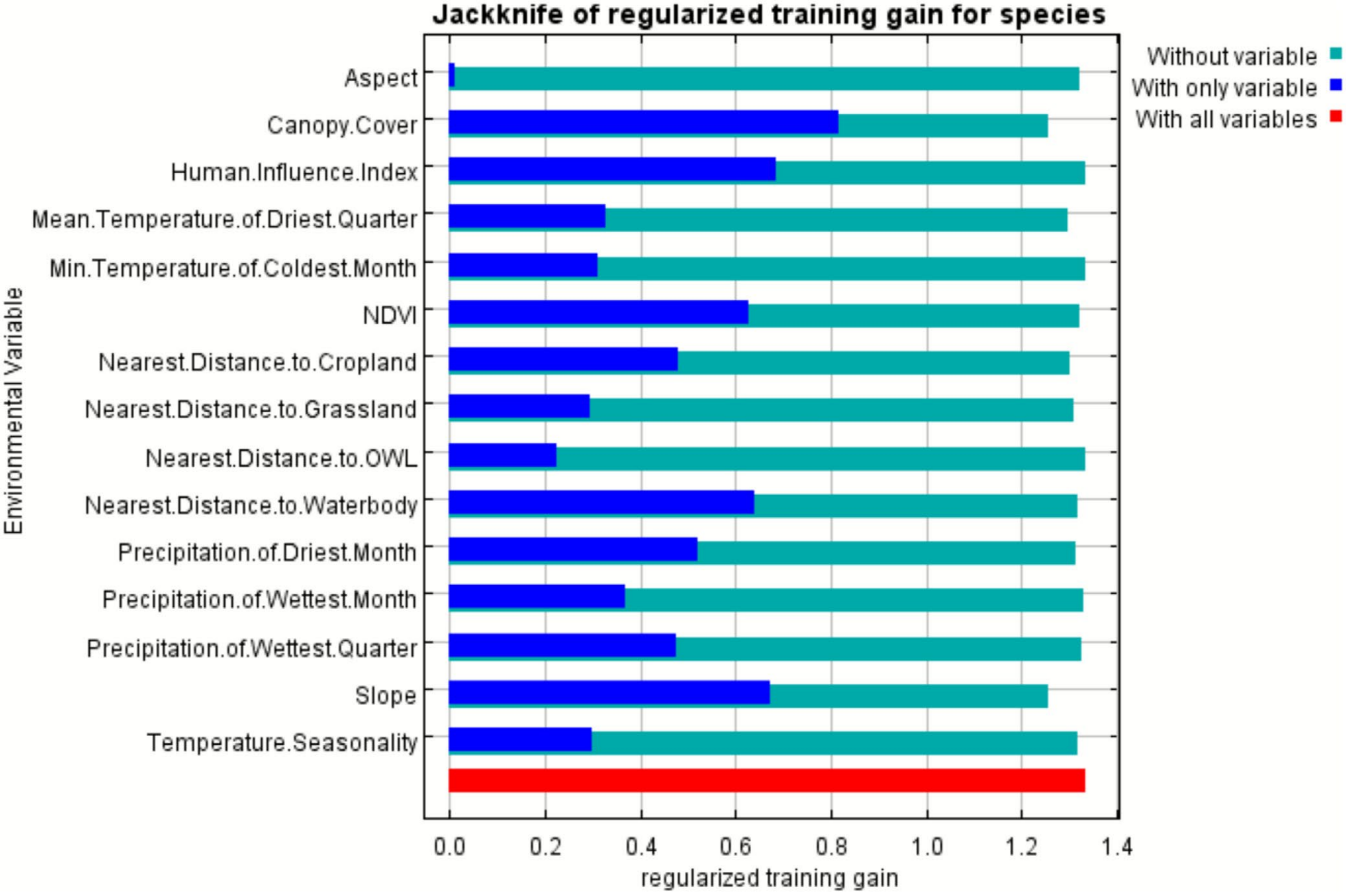
The importance of predictor variables in the maxent model of human–leopard conflict shown by the jackknife test, where the aqua, blue, and red bars represent the results without the variable, with only the variable, and with all variables, respectively.

The results highlighted the influence of anthropogenic pressures, habitat structure, and climatic factors, with variables such as canopy cover, the HII, slope, and proximity to a waterbody being particularly important in determining conflict occurrence, as confirmed by the jackknife test. Furthermore, bioclimatic factors, along with land‐use characteristics such as precipitation, NDVI, and cropland proximity, contributed moderately to the model's predictive performance, emphasizing the need to consider landscape features, agricultural expansion, and leopard habitat availability when assessing conflict scenarios (Figure 4).

### 3.3 People's Attitudes Towards Leopard Conservation

**FIGURE 5:**
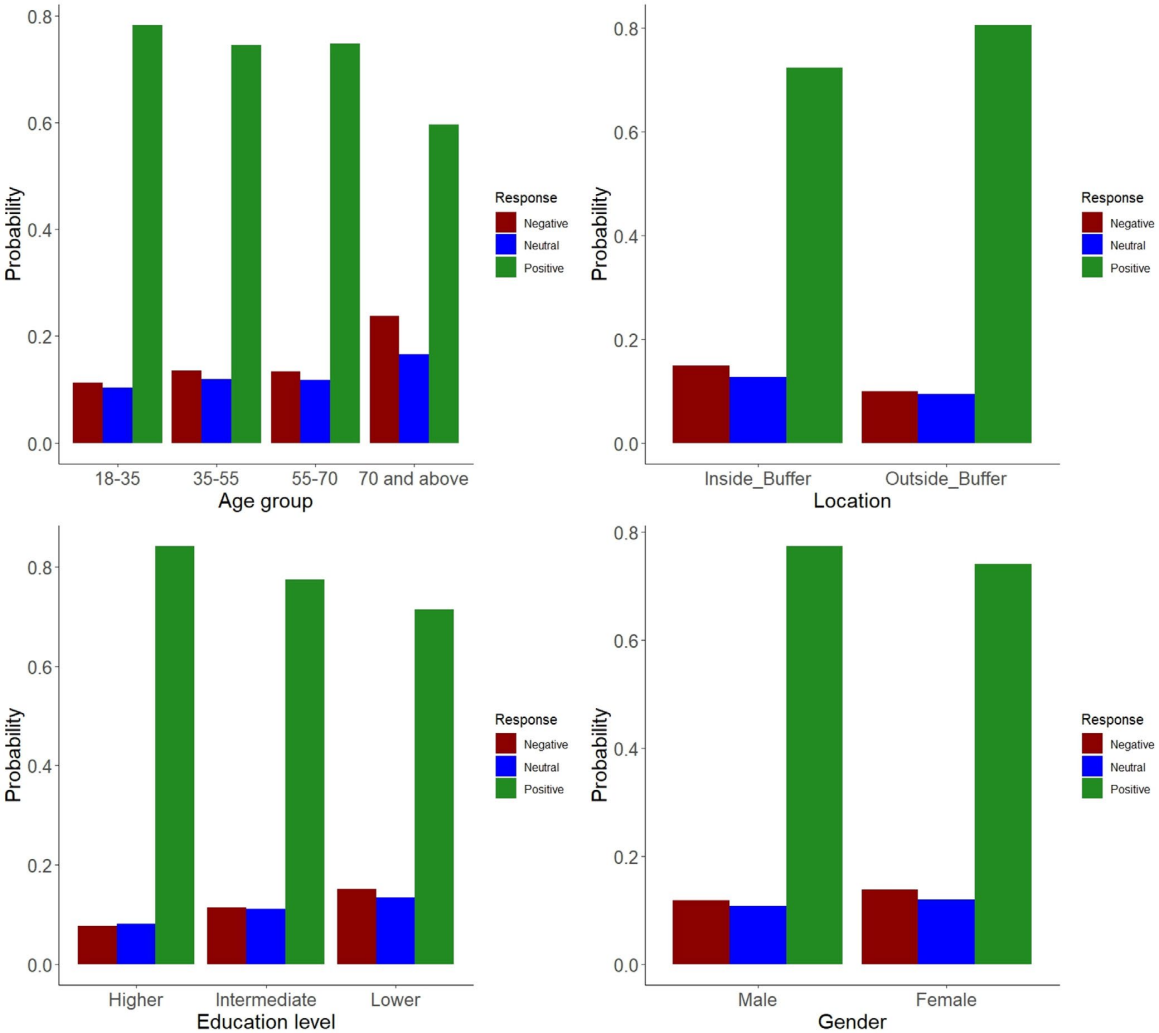
Perception of local people towards the human–leopard conflict based on an individual level (i.e., age, location, education level, and gender).

Survey responses (Table S1) showed overall a positive attitude of people towards leopard conservation. However, the attitudes varied based on demographics at the individual levels (Figure 5). Males were slightly more likely to have a positive attitude towards leopard conservation than females, but the effect is weak and not statistically significant (Coef = 0.175, OR = 1.19, *p* = 0.261). The odds of people living outside the buffer zone, where human–leopard interactions are less frequent, having more favorable attitudes towards leopards were 1.62 times higher than those living inside the buffer zone (Coef = 0.485, OR = 1.62, *p* = 0.001), highlighting the importance of residential location in shaping people's attitudes (*χ* ^2^(2) = 10.221, *p* = 0.001). Similarly, educational background also played a crucial role (*χ* ^2^(2) = 14.324, *p* < 0.001); respondents with intermediate (Coef = −0.433, OR = 0.65, *p* = 0.036) or lower education (Coef = −0.748, OR = 0.47, *p* < 0.001) were less likely to support leopard conservation than those with higher education, indicating a decline in positive attitudes with decreasing education level. In contrast, age had a less pronounced impact, with only individuals over 70 years old showing significantly negative attitudes (Coef = −0.895, *p* = 0.038). The highly significant (*p* < 0.001) threshold coefficients indicate that altering perceptions of leopards from negative to neutral and from neutral to positive is unlikely (Table 2).

**TABLE 2:** Ordinal logistic regression analysis of attitudes towards leopard conservation. The model estimates the odds of a respondent reporting a more positive attitude (e.g., shifting from “negative” to “neutral” or “neutral” to “positive”). Odds Ratios (OR) greater than 1 indicate increased odds of a more positive attitude, while values less than 1 indicate decreased odds, relative to the reference category. Reference categories were Female (Gender), Inside buffer zone (Location), Higher education (Education), and Age 18–35 (Age Group).

Variable	*β* (Coef)	SE	*t*‐value	*p*‐value	Odds ratio (exp(*β*))	95% CI for odds ratio
Gender (Ref: Female)						
Male	0.175	0.144	1.125	0.261	1.19	0.90–1.57
Intercept(negative|neutral)	−1.828	0.123	−14.879	< 0.001**		
Intercept(neutral|positive)	−1.053	0.109	−9.653	< 0.001**		
Location (Ref: Inside buffer)						
Outside buffer zone	0.485	0.146	3.331	0.001**	1.62	1.21–2.16
Intercept(negative|neutral)	−1.272	0.109	−15.810	< 0.001**		
Intercept(neutral|positive)	−0.940	0.094	−10.039	< 0.001**		
Education (Ref: Higher education)						
Intermediate education	−0.433	0.206	−2.096	0.036*	0.65	0.43–0.97
Lower education	−0.748	0.203	−3.683	< 0.001**	0.47	0.32–0.69
Intercept(negative|neutral)	−2.474	0.179	−13.804	< 0.001**		
Intercept(neutral|positive)	−1.664	0.166	−10.033	< 0.001**		
Age group (ref: 18–35)						
Age 35–55	−0.207	0.156	−1.331	0.183	0.81	0.60–1.09
Age 55–70	−0.196	0.224	−0.871	0.384	0.82	0.53–1.27
Age > 70	−0.895	0.432	−2.072	0.038*	0.41	0.17–0.97
Intercept(negative|neutral)	−2.063	0.124	−16.593	< 0.001**		
Intercept(neutral|positive)	−1.285	0.109	−11.76	< 0.001**		

* Significant at *α* = 0.05.

** Significant at *α* = 0.01.

## 4 Discussion

### 4.1 Spatial Patterns and Factors of HLC in Kathmandu Valley

Our study indicated variations in the spatial distribution of HLC hotspots across the Kathmandu Valley. The analysis revealed that about 0.91% of the area falls within the high HLC risk zone, much of which corresponds to forest‐edge landscapes and recently developed peri‐urban zones. These areas include the buffer zones of Shivapuri Nagarjun National Park (SNNP), as well as the settlements in regions like Kirtipur, Chandragiri, and the eastern part of Kathmandu municipality, where recent and rapid settlement expansion has contributed to higher conflict probabilities. These findings align with previous studies (Maharjan 2017; Bista et al. 2022; Manandhar et al. 2025), which similarly reported increased human–leopard interactions and predation incidents in peri‐urban and forest‐edge areas driven by settlement expansion and habitat fragmentation around Kathmandu.

Based on the jackknife test, the analysis of top factors revealed that a combination of anthropogenic, topographic, and geographic variables contributes to HLC. These findings are consistent with broader research in Nepal's mid‐hills, which shows that leopard attack occurrence is strongly and positively associated with human population density and terrain ruggedness (Poudel et al. 2023). Among these, canopy cover and the HII had important contributions to the model.

Leopards are highly adaptable and often use habitats near forest edges, which serve as corridors for their daily movements between hunting grounds and resting sites (Ngoprasert et al. 2007; Lamichhane et al. 2019; Bista and Song 2022). While vegetation cover and human disturbances play major roles in determining habitat suitability (Kshettry et al. 2017; Zeng et al. 2022; Majumder et al. 2024), leopards can adapt themselves near human settlements (Athreya and Belsare 2007) facilitated by the extent of vegetative cover to ambush and prey (Athreya et al. 2004, 2007; Karki and Rawat 2014; Athreya et al. 2015). When the forest cover is reduced, their natural hiding spots diminish, thus increasing their visibility and the likelihood of encounters with humans. This heightened visibility combined with greater human pressure on wildlife leads to a higher likelihood of conflict (Athreya et al. 2013; Bharali et al. 2020).

Leopards, like other carnivores, being sensitive to human presence may adopt nocturnal behaviors near forest edges or settlement areas to avoid human interactions (Sunquist 1981; Griffiths and van Schaik 1993; Ngoprasert et al. 2007; Odden et al. 2014). In areas where natural prey is scarce, leopards are often drawn to human settlements, where livestock and stray dogs provide an easily accessible food source (Poudel et al. 2023). Scat analyses from different parts of Nepal confirm that when natural prey is depleted, leopards increasingly rely on domestic animals, which can lead to more frequent HLCs (Dhungana et al. 2019; Kandel et al. 2020; Lamichhane et al. 2019). This combination of behavioral adaptation and prey availability near settlement areas explains why most livestock and stray dog depredation incidents in the study area occur at night (Odden et al. 2014; Maharjan 2017; Manandhar et al. 2025).

Other important environmental factors affecting the occurrence of human–wildlife conflict include slope and proximity to a water body, which are often visited by carnivores and other prey species (Maharjan et al. 2024). Previous studies (Athreya et al. 2013; Mondal et al. 2013; Ramesh et al. 2020; Rather et al. 2020; Majumder et al. 2024) indicate that leopards often prefer areas with gentle slopes, intermediate cover, and near water sources for preying upon domesticated animals, indicating a higher potential for human–leopard interactions and conflict in those areas. Moreover, increasing human encroachment into leopard habitats may contribute to heightened conflict in areas with gentle slopes.

### 4.2 People's Attitudes and Conservation Implications

Conflict incidents in the Kathmandu valley primarily involved attacks on both humans and livestock near human settlements and forest edges, similar to previous studies in Nepal (Athreya et al. 2007; Thapa 2014; Athreya et al. 2015; Koirala et al. 2012; Karki and Rawat 2014). Despite this severity, the attitude of the people towards leopards was positive, consistent with the previous findings of the study done in the valley (Bhandari and Thapa 2015) but differing from the studies in Annapurna Conservation Area (ACA), Nepal (Koirala et al. 2012), and the forest of Pakistan (Kabir et al. 2014). The contradictions in the results could be based on the varying degrees of exposure to conflict and economic dependence on livestock. In regions where leopard‐related losses are frequent and severe, people's attitudes tend to be more negative (Suryawanshi et al. 2014).

Additionally, this study showed that the socio‐demographic factors, such as education level and location of the settlement, significantly influenced perceptions. The role of socio‐demographic factors in shaping these perceptions may be due to the farsightedness for balancing the avoidance of unforeseen threats with conservation concerns (Lagendijk and Gusset 2008; Kansky and Knight 2014; Suryawanshi et al. 2014). For instance, educated people with less exposure to the loss are more likely to support conservation; whereas, the frequent presence of large carnivores tends to provoke a negative attitude, which may diminish over time with increased exposure (Zimmermann et al. 2001; Bhandari et al. 2019). The findings support the current results, as the presence of leopards in a human‐dominated landscape of the Kathmandu Valley has been witnessed for more than a decade.

The increasing trend of the HLC over the years underscores the need for intervention, as fragmented forest patches and rapid human encroachment are likely to aggravate the issue if left unaddressed. In Nepal, the government provides financial compensation for damages and casualties involving humans or livestock caused by leopards and other predators (DNPWC 2023). However, for long‐term coexistence, a more proactive approach is necessary. Implementing strategies such as building animal corridors, encouraging community‐based conflict resolution, and raising knowledge about time‐restricted movement in high‐risk locations could all help to decrease fatal interactions. To maintain and enhance positive attitudes towards leopards, awareness programs should target specific groups, such as individuals with lower to intermediate educational backgrounds and those residing within buffer zones. Such actions would not only improve human safety but would also contribute to a positive public perception of leopards, promoting a more sustainable approach to conservation.

Since this study largely relied on available secondary data, many minor conflict cases may have gone unnoticed or unreported over the years. Moreover, our model was based solely on conflict locations; future studies should focus on severity‐based conflict mapping by assigning weights to different conflict types.

## 5 Conclusion

This study provides valuable insights into the spatial distribution and key drivers of HLC in the Kathmandu Valley. The findings of the study indicate that conflict hotspots range from the edge of the forests to the core of the city, with key factors including canopy cover, human disturbance, slope, and proximity to water bodies. Despite the increasing decadal trends of conflicts, people have a positive attitude towards leopard conservation and tend to have a great tolerance for the presence of leopards in their settlements. At an individual level, attitude is affected by education level and the location of the respondents.

In a valley like Kathmandu, there is no such flexibility to locate settlements at a safer distance from the forest edge. Given the recent increase in conflict, measures to mitigate HLC and maintain coexistence in areas where forests and human‐dominated landscapes intersect are considered necessary. A coexistence model could be developed using robust HLC models, incorporating potential distribution patterns and identifying key variables influencing conflicts in a mosaic of forest and settlements. Additionally, awareness training for residents near forested areas would help them better cope with situations where leopards enter their settlements.

## Author Contributions

**Pratistha Shrestha:** data curation (equal), formal analysis (equal), methodology (equal), software (equal), visualization (equal), writing – original draft (lead), writing – review and editing (equal). **Khem Lal Bishwakarma:** conceptualization (lead), data curation (equal), methodology (equal), validation (equal), visualization (equal), writing – original draft (supporting), writing – review and editing (equal). **Rupesh Maharjan:** data curation (supporting), methodology (equal), validation (equal), visualization (equal), writing – review and editing (equal). **Dipesh Kumar Sharma:** conceptualization (supporting), data curation (equal), formal analysis (equal), methodology (equal), visualization (equal), writing – original draft (supporting), writing – review and editing (equal).

## Conflicts of Interest

The authors declare no conflicts of interest.

## Supporting information


**Table S1:** People's attitude scores towards the leopard conservation questions in Kathmandu Valley.

## Data Availability

Data are available at the Github Repository: https://github.com/DipeshDFRS/HLC.
