# Supplementary material for: Claws in the Capital: Human–Leopard Conflict Hotspots and Community Perceptions in Kathmandu Valley, Nepal
Source: Ecol Evol. 2025 Dec 16;15(12):e72678. doi: 10.1002/ece3.72678 (PMC12706532; doi:10.1002/ece3.72678)
Supplement: Supplementary file 1 — Table S1: People's attitude scores towards the leopard conservation questions in Kathmandu Valley. [file ECE3-15-e72678-s001.docx]

Table S1. People’s attitude scores towards the leopard conservation questions in Kathmandu Valley

| Questions | Possible reply | Score | Response (%) |
| --- | --- | --- | --- |
| 1. Would you like to conserve this animal? | Yes | 1 | 86.5 |
|  | No | -1 | 7.0 |
|  | Don’t know | 0 | 6.5 |
| 2. Do you think the conservation of this animal is beneficial for the environment? | Yes | 1 | 91.4 |
|  | No | -1 | 2.2 |
|  | Don’t know | 0 | 6.5 |
| 3. Should you teach your kids about leopard conservation? | Yes | 1 | 82.7 |
|  | No | -1 | 1.6 |
|  | Don’t know | 0 | 15.7 |
| 4. Where should leopards be protected? | National Park | 1 | 38.9 |
|  | Don’t know | 0 | 3.2 |
|  |  |  |  |
|  | Zoo | -1 | 57.8 |
| 5. What should be done when leopards enter a settlement? | Inform forest office | 1 | 41.4 |
|  | Stay away | 0 | 21.6 |
|  | Bear livestock loss | 2 | 3.5 |
|  | Do nothing | 0 | 3.2 |
|  | Chase them away | -1 | 25.9 |
|  | Kill | -2 | 4.3 |
